# E2F4 may be a core transcription factor in the lncRNA‐TF regulatory network in cervical cancer

**DOI:** 10.1002/jcla.24322

**Published:** 2022-03-09

**Authors:** Haiyan Yang, Xiaoli Qu, Jiangang Huang, Fan Zhang, Ziyu Fang, Bin Zhao, Yifeng Wang

**Affiliations:** ^1^ Department of Obstetrics Gynecology Zhujiang Hospital Southern Medical University Guangzhou China; ^2^ Department of Gynecological Oncology The Fourth Affiliated Hospital of Guangxi Medical University Liuzhou China; ^3^ Department of Gynecological Oncology The Fifth Affiliated Hospital of Guangxi Medical University Liuzhou China; ^4^ 12466 Fujian Provincial Key Laboratory of Innovative Drug Target Research and State Key Laboratory of Cellular Stress Biology School of Pharmaceutical Sciences Xiamen University Xiamen China; ^5^ Shanghai OE Biotech Co., Ltd Shanghai China; ^6^ Xiamen Health and Medical Big Data Center Xiamen China

**Keywords:** cervical cancer, LncRNA, microarray, transcription factor

## Abstract

**Background:**

Cervical cancer is the most common gynecological cancer worldwide and is associated with high morbidity and mortality. Despite improvements in therapeutic strategies, the network regulation mechanism remains unclear and the treatment effect is not satisfactory. Therefore, there is a need to continue studying the mechanism of cervical cancer to explore effective gene targets and precise targeted therapy drugs.

**Methods:**

First, three paired tissues (cancer tissues and noncancerous tissues) from patients with cervical squamous cell carcinoma were collected, grouped, and analyzed by microarray. Second, differentially expressed mRNAs (DEMs) and differentially expressed lncRNAs (DELs) (|fold change| ≥ 2 and *p* < 0.05) between the two groups were screened. For DEMs, functional annotation and pathway analysis were performed using DAVID. Functional prediction of DELs was then performed and their cis‐regulatory and trans‐regulatory networks were explored.

**Results:**

Function prediction of DELs (both up‐regulated and down‐regulated) shows that the highest frequency Cellular Component (CC) item is cytosol, the highest frequency Molecular function (MF) item is mitotic cell cycle and the highest frequency Biological Process (BP) item is protein binding. Through cis‐regulation analysis of DELs, the cis‐regulatory relationship of 96 DELs was predicted. The lncRNA‐trans‐regulation network analysis suggested that E2F4 may be the core transcription factor in the lncRNA‐TF regulatory network in cervical cancer.

**Conclusions:**

The lncRNA‐TF regulatory network plays an important role in the occurrence and progression of cervical cancer, and E2F4 may be a critical transcription factor in the regulatory network.

## Graphical abstract

We found the differently expressed lncRNAs between cervical cancer tissues and adjacent noncancerous tissues. Through the prediction of lncRNAs function, cis‐regulation of lncRNA and trans‐regulation of lncRNA, we construct lncRNA‐TF regulatory network and found that E2F4 may be the potential critical transcription factor in the regulatory network.

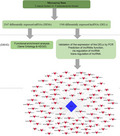

## 1 INTRODUCTION

Although the level of cervical cancer screening and HPV infection prevention and control is gradually being emphasized and improved, cervical cancer still has high morbidity and high mortality, and is still the most common gynecological cancer worldwide. Globally, there were 604,127 new cases and 341,831 deaths from cervical cancer in 2020.^1^ Both the incidence and mortality rates of cervical cancer are ranked fourth among malignancies in women.^1^ Its annual rate is 16.56 per 100,000 people, and the mortality rate is as high as 5.04 per 100,000 people.^2^ According to the American Cancer Society, there are 14,480 new diagnoses and more than 4290 deaths from cervical cancer in 2021 in the USA.^3^ The traditional treatment for cervical cancer is surgery combined with radiotherapy, chemotherapy or radical radiotherapy. For patients with FIGO staging of IB to IIA without lymph node metastasis, the five‐year survival rate can reach 88%–95%.^4^ However, advanced patients have no opportunity for surgery, the lesion may not be removed completely for those with lymph node metastasis, and relapsed and uncontrolled patients face the problem of drug resistance. Therefore, the efficacy of traditional treatment for patients with the advanced and relapsed disease is unsatisfactory. The 5‐year survival rate of patients with stage IV disease is less than 15%. Molecular targeted therapy provides new ideas for the treatment of these patients. The search for effective markers is of great significance for perfecting molecular subtype classification and molecular targeted therapy for cervical cancer. Approximately 76% of the human genome is transcribed as lncRNA, which is longer than 200 nucleotides and has a structure more complex than mRNAs.^5^, ^6^, ^7^, ^8^ The versatility of the lncRNA structure enables it to bind to target molecules and participate in the regulation of gene expression in a variety of ways at the epigenetic, transcriptional, and posttranscriptional levels, contributing to the occurrence and development of various human diseases, especially apoptosis, invasion, and metastasis of various tumors.^9^

In recent years, studies have found that some lncRNAs, such as HOTAIR,^10^ MALAT1,^11^ PVT1,^12^ H19,^13^ and XIST,^14^ play important regulatory roles in cell proliferation, invasion, and migration in cervical cancer.

## 2 MATERIALS AND METHODS

### 2.1 RNA isolation, labeling, and microarray hybridization

Total RNA of three paired tissues (cancer tissues and noncancerous tissues) from patients with cervical squamous cell carcinoma was extracted using TRIzol reagent (Invitrogen) and quantified using the NanoDrop ND‐2000 (Thermo Scientific). RNA integrity was then assessed using an Agilent Bioanalyzer 2100 (Agilent Technologies). After confirming that the quality inspection was qualified, sample labeling, chip hybridization, and elution were performed based on the manufacturer's standard protocols. First, total RNA was reverse transcribed into double‐stranded cDNA, and then cRNA labeled with Cyanine‐3‐CTP (Cy3) was synthesized. The labeled cRNA was hybridized with the chip and the original image was obtained by scanning with an Agilent Scanner G2505C after elution.

### 2.2 Microarray data analysis

Feature extraction software (version 10.7.1.1, Agilent Technologies) was used to process the original image and extract the original data. Then, the original data were imported into Genespring software (version 14.8, Agilent Technologies) for quantile standardization and data filtering. At least one set of 100% probes marked with “P” in each set of samples used for comparison was left for subsequent analysis. Differentially expressed mRNAs/lncRNAs were identified through fold‐changes. The threshold criteria for up‐ and down‐regulated signatures is the absolute value of a fold change of more than or equal to 2.0, and *p* < 0.05. (Please refer to file in [Link], [Link] (Cancer vs. Noncancer differentially expressed mRNA coding.txt) and (Cancer vs. Noncancer differentially expressed lncRNA nocoding.txt)).

### 2.3 Functional enrichment analysis of differentially expressed signatures

Gene Ontology (GO) and Kyoto Encyclopedia of Genes and Genomes (KEGG) analyses^15^ were applied to determine the roles of these DEMs in an open‐access database DAVID (https://david.ncifcrf.gov/summary.jsp), which is used for annotation, visualization, and integrated discovery.^16^ In addition, the function of DELs was indirectly predicted by annotation of differentially expressed mRNAs that were co‐expressed with DELs.

### 2.4 Construction of co‐expression network of lncRNA‐mRNA

The Pearson correlation coefficient (PCC) was calculated to evaluate the co‐expression relationships between the DELs and DEMs. The co‐expressed signatures with PCC > 0.7 or <−0.7 and a correlation *p* value < 0.05 were identified as statistically significant.^17^

### 2.5 Prediction of lncRNAs function

For functional prediction of lncRNAs, GO, and KEGG pathway enrichment analyses were performed for the co‐expressed genes of DELs using cluster Profiler 3.0.1 in R. The enriched functional terms of the co‐expressed genes of the DELs were used as the predicted functions of the DELs. The hypergeometric cumulative distribution function was used to calculate the enrichment of functional terms in the annotation of the co‐expressed genes. The false discovery rate was calculated using the default.

### 2.6 Cis‐regulation of lncRNAs

All coding genes within 100k upstream and downstream of DELs were searched and the intersection of genes that were significantly co‐expressed with DELs was determined. These genes, which are adjacent to the genome and co‐expressed in their expression patterns, are likely to be regulated by lncRNAs; hence, they were identified as potential “cis‐regulated lncRNAs”.^18^

### 2.7 Trans‐regulation factors of lncRNAs

*The co*‐*expression network of lncRNA*‐*mRNA screened by PCC and p value was constructed*, *and the transcription factor*‐*target gene relationship pairs were retrieved through the GTRD database* (https://gtrd.biouml.org/).^19^ DEMs co‐expressed with DELs were screened when these DEMs were matched to the target gene set of transcription factors (TFs). Significance was calculated using a hypergeometric cumulative distribution function. The top 100 co‐expression pairs of lncRNA‐TF and the top 10 lncRNA‐TF‐mRNAs were selected for the construction of the regulatory network, which was visualized using Cytoscape 3.3.0 (available at http://www.cytoscape.org/).

### 2.8 Statistical analysis

The moderate t‐test was used to identify DEMs and DELs. Fisher's exact test was applied to perform GO and KEGG analysis. All statistical analyses were carried out in R 4.0.1 software.

## 3 RESULTS

### 3.1 Overview of lncRNA and mRNA profiles in cervical cancer and adjacent noncancerous tissues

**FIGURE 1:**
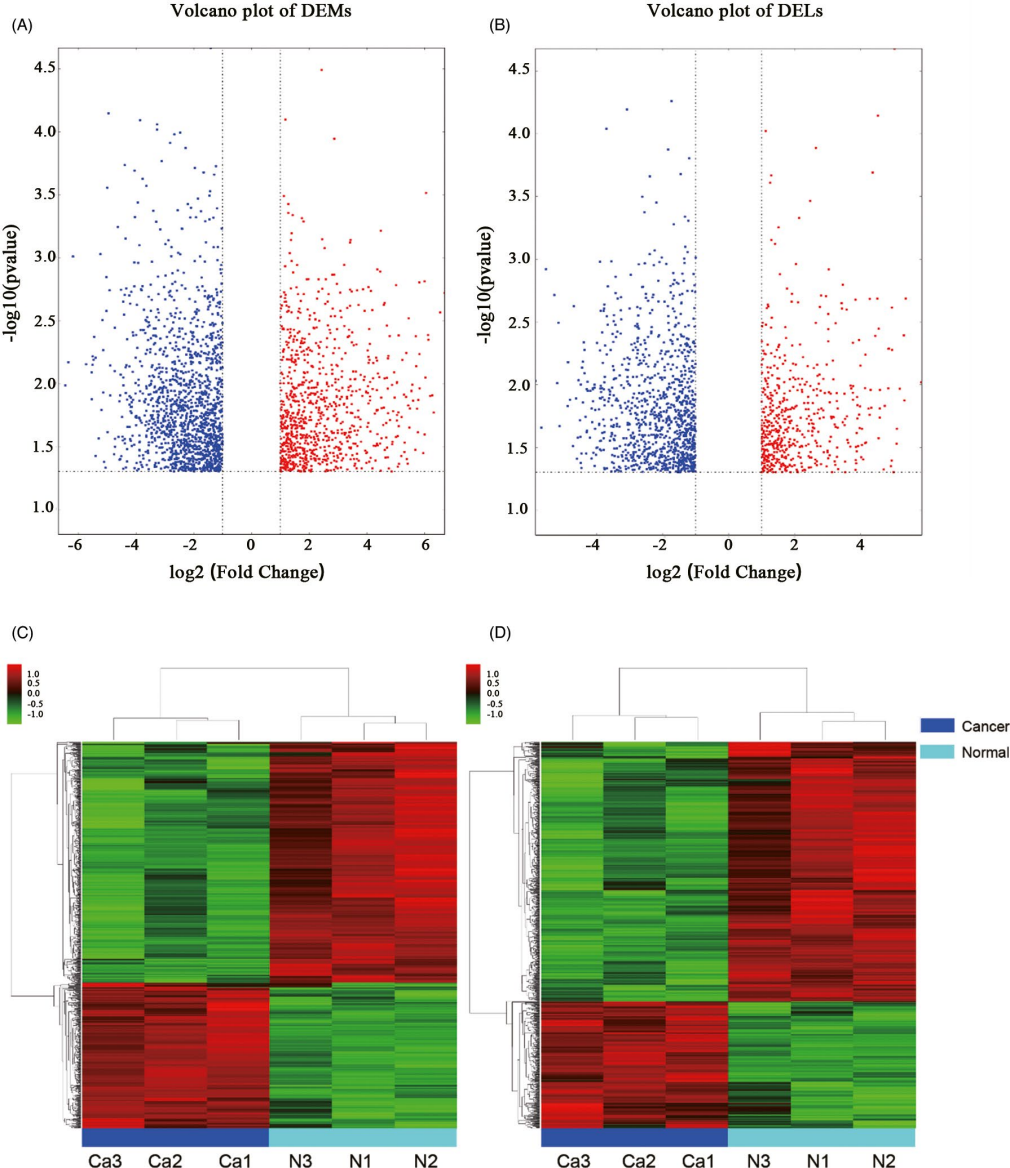
Volcano plot and hierarchical clustering analysis of differentially expressed genes between cervical cancer and adjacent normal tissues. Red color indicates an upregulation of transcripts and green color indicates transcripts that are downregulated. (A) Volcano plot of differentially expressed mRNAs. (B) Volcano plot of differentially expressed lncRNAs. (C) Hierarchical clustering analysis of differentially expressed mRNAs. (D) Hierarchical clustering analysis of differentially expressed lncRNAs

**FIGURE 2:**
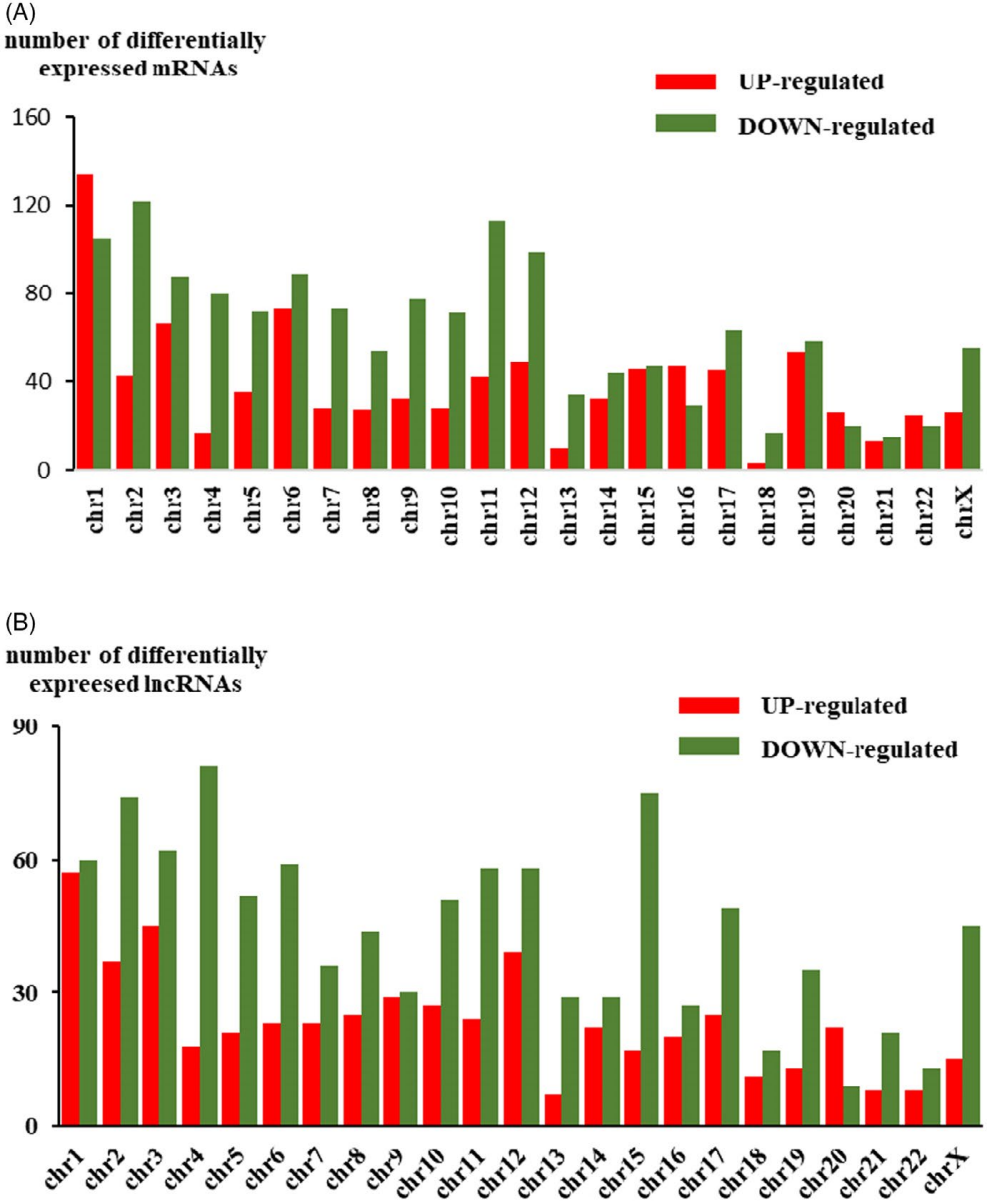
Distribution of differentially expressed lncRNAs and mRNAs on each chromosome. (A) The distribution of upregulated and downregulated mRNAs on each chromosome. (B) The distribution of upregulated and downregulated lncRNAs on each chromosome. lncRNA: long noncoding RNA; chr: chromosome

The Agilent Human lncRNA V6 (4*180K, Design ID: 084410) chip which contains 37549 mRNA probes, 79404 lncRNA probes was used in this project. After using DESeq2 analysis, 2347 DEMs were obtained, of which 901 were upregulated DEMs and 1446 were downregulated DEMs. For DELs, 1568 were obtained, of which 543 were upregulated DELs and 1025 were downregulated DELs compared with those in noncancerous tissues. (Table 1). Among the DELs the most upregulated 10 genes were BIRC5, NEK2, CDCA2, TOP2A, CDKN3, ASPM, SYCP2, CDC20, SERPINB4, and CLSPN, while the most down‐regulated 10 genes were DMD, MYH11, DMD, RSPO3, MYOCD, FHL1, PWAR5, LPHN3, STON1‐GTF2A1L, and CHRDL2. Volcano plots of DEMs and DELs were used to plot the absolute value of fold changes and p‐values against each other between cancer samples and noncancerous samples (Figure 1A,B). DELs and DEMs were used to perform hierarchical cluster analysis of the three paired samples (Figure 1C,D). The differentially expressed genes were not equally distributed on each chromosome (Figure 2A,B).

**TABLE 1:** The differentially expressed Genes in cervical cancer tissues and adjacent noncancerous tissues

Type	Number	Up‐regulated	Down‐regulated
mRNA	2347	901	1446
LncRNA	1568	543	1025
Total	3915	1444	2471

### 3.2 GO and KEGG pathway analysis of differentially expressed coding genes

**FIGURE 3:**
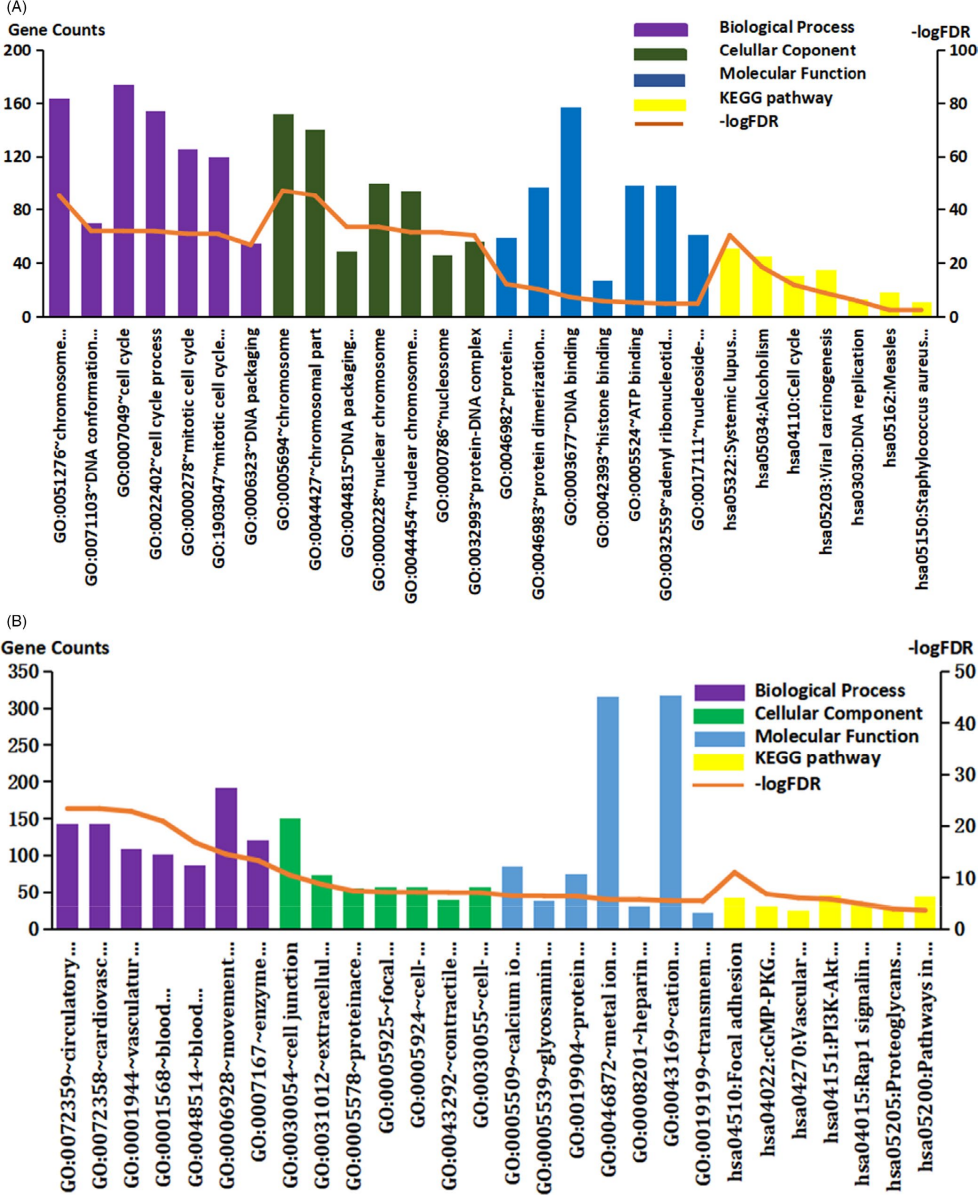
The top seven GO and KEGG annotation of DEMs. (A) The top seven GO and KEGG annotation of up‐regulated DEMs. (B) The top seven GO and KEGG annotation of down‐regulated DEMs

As shown in Figure 3A,B, and Table 2, the upregulated DEMs are mainly located on the chromosome and play a role in the cell cycle, DNA replication, and carcinogenesis by binding to DNA and ATP. On the other hand, the downregulated genes are mainly located in the extracellular matrix and cell junctions, and participate in various signaling pathways, such as the cGMP‐PKG signaling pathway and PI3K‐Akt signaling pathway, through binding to protein domains, metal ions, and glycosaminoglycans, thereby affecting adhesion and angiogenesis.

**TABLE 2:** The top five GO and KEGG enrichment terms of DEMs

Category	Term	Description	Count	Fold Enrichment	FDR
*Up‐regulated DEMs*					
BP	GO:0051276	Chromosome organization	164	3.597893916	4.91E‐46
BP	GO:0071103	DNA conformation change	70	6.402298345	1.06E‐32
BP	GO:0007049	Cell cycle	174	2.703144011	1.17E‐32
BP	GO:0022402	Cell cycle process	154	2.947793667	1.36E‐32
BP	GO:0000278	Mitotic cell cycle	126	3.381048465	1.30E‐31
CC	GO:0005694	Chromosome	152	3.882319225	7.28E‐48
CC	GO:0044427	Chromosomal part	140	4.051443358	4.35E‐46
CC	GO:0044815	DNA packaging complex	49	10.37779009	3.34E‐34
CC	GO:0000228	Nuclear chromosome	100	4.312153999	3.47E‐34
CC	GO:0044454	Nuclear chromosome part	94	4.33458717	3.27E‐32
MF	GO:0046982	Protein heterodimerization activity	59	3.357051742	8.13E‐13
MF	GO:0046983	Protein dimerization activity	97	2.212446213	6.76E‐11
MF	GO:0003677	DNA binding	157	1.621360182	6.08E‐08
MF	GO:0042393	Histone binding	27	3.861455301	1.90E‐06
MF	GO:0005524	ATP binding	99	1.752076151	6.67E‐06
KEGG	hsa05322	Systemic lupus erythematosus	51	8.233103351	4.38E‐31
KEGG	hsa05034	Alcoholism	45	5.499680205	3.11E‐19
KEGG	hsa04110	Cell cycle	31	5.408018868	1.55E‐12
KEGG	hsa05203	Viral carcinogenesis	35	3.693281178	2.11E‐09
KEGG	hsa03030	DNA replication	13	7.811582809	1.47E‐06
*Down‐regulated DEMs*					
BP	GO:0072359	Circulatory system development	143	2.649341545	4.27E‐24
BP	GO:0072358	Cardiovascular system development	143	2.649341545	4.27E‐24
BP	GO:0001944	Vasculature development	109	3.128458396	1.47E‐23
BP	GO:0001568	Blood vessel development	102	3.10035489	1.23E‐21
BP	GO:0048514	Blood vessel morphogenesis	86	3.066554447	1.49E‐17
CC	GO:0030054	Cell junction	150	1.85248158	3.15E‐11
CC	GO:0031012	Extracellular matrix	74	2.377840695	1.92E‐09
CC	GO:0005578	Proteinaceous extracellular matrix	55	2.594671568	3.86E‐08
CC	GO:0005925	Focal adhesion	57	2.482704341	7.14E‐08
CC	GO:0005924	Cell‐substrate adherens junction	57	2.463800501	7.65E‐08
MF	GO:0005509	Calcium ion binding	85	2.057073256	3.51E‐07
MF	GO:0005539	Glycosaminoglycan binding	38	3.1700802	4.13E‐07
MF	GO:0019904	Protein domain specific binding	75	2.119546165	4.13E‐07
MF	GO:0046872	Metal ion binding	316	1.31618763	2.02E‐06
MF	GO:0008201	Heparin binding	31	3.361953475	2.02E‐06
KEGG	hsa04510	Focal adhesion	43	3.788674847	9.48E‐12
KEGG	hsa04022	cGMP‐PKG signaling pathway	31	3.561153602	1.50E‐07
KEGG	hsa04270	Vascular smooth muscle contraction	25	3.878289696	9.66E‐07
KEGG	hsa04151	PI3K‐Akt signaling pathway	46	2.42005277	1.76E‐06
KEGG	hsa04015	Rap1 signaling pathway	32	2.765774595	1.46E‐05

### 3.3 Function prediction of lncRNAs

**FIGURE 4:**
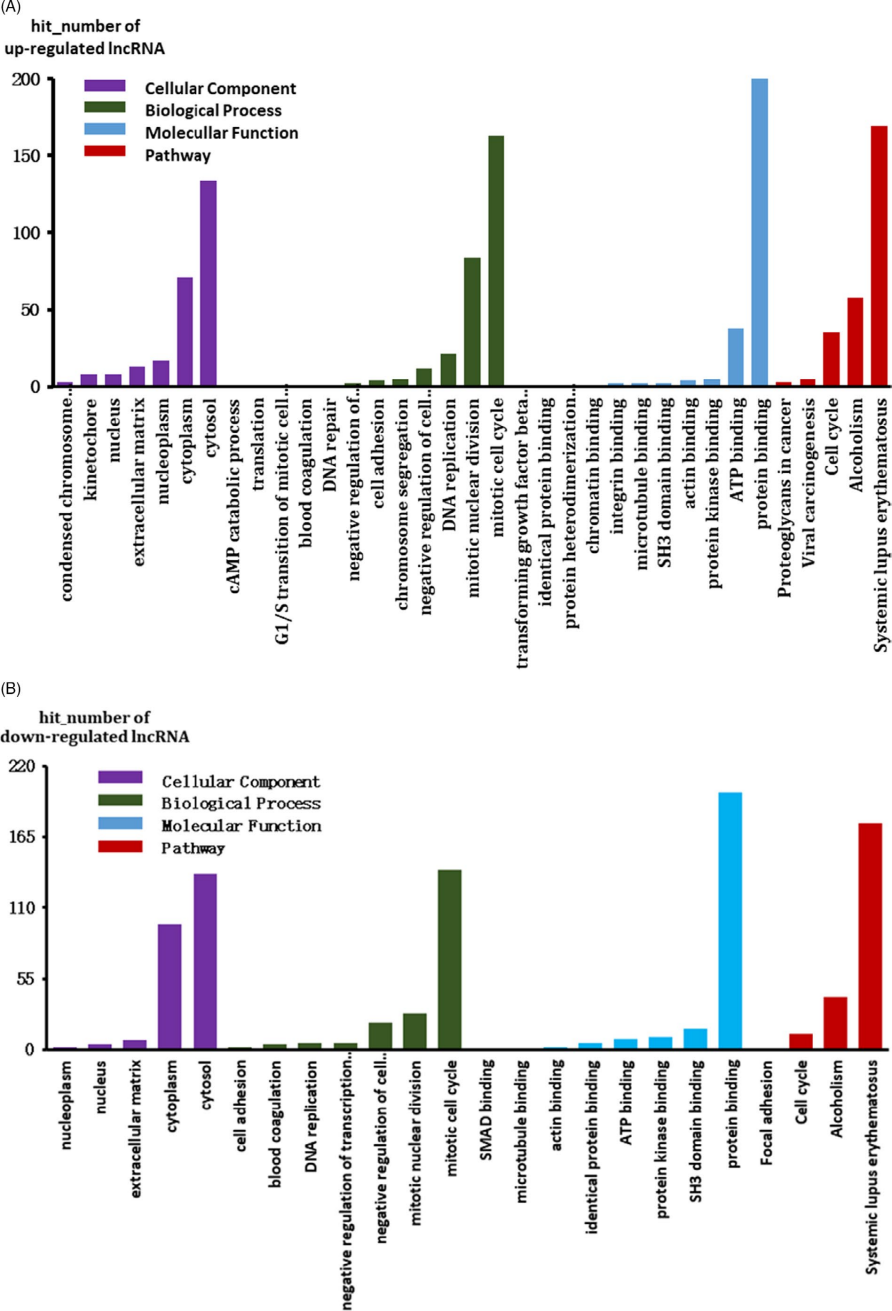
Function prediction of DELs. (A) Function prediction of up‐regulated DELs. (B) Function prediction of down‐regulated DELs

For DELs, the top 500 prediction relationships with the highest prediction reliability (sorted by *p*‐value) were selected and the frequency of each prediction item was counted. Enriched functional items as the predictive function of each DEL were used. The hypergeometric cumulative distribution function was used to calculate the enrichment of functional terms in co‐expressed genes. The hit numbers for each item are shown in Figure 4A,B. The three items with the highest hit numbers are listed in Table 3.

**TABLE 3:** The top three GO and KEGG enrichment terms of the DELs hit

Category	Term	Description	Count
*Up‐regulated LncRNAs*			
CC	GO:0005829	Cytosol	134
	GO:0005737	Cytoplasm	71
	GO:0005654	Nucleoplasm	17
BP	GO:0000278	Mitotic cell cycle	163
	GO:0007067	Mitotic nuclear division	84
	GO:0006260	DNA replication	21
MF	GO:0005515	Protein binding	200
	GO:0005524	ATP binding	38
	GO:0019901	Protein kinase binding	5
Pathway	hsa05322	Systemic lupus erythematosus	169
	hsa05034	Alcoholism	58
	hsa04110	Cell cycle	35
*Down‐regulated LncRNAs*			
CC	GO:0005829	Cytosol	136
	GO:0005737	Cytoplasm	97
	GO:0031012	Extracellular matrix	7
BP	GO:0000278	Mitotic cell cycle	139
	GO:0007067	Mitotic nuclear division	28
	GO:0008285	Negative regulation of cell proliferation	21
MF	GO:0005515	Protein binding	200
	GO:0017124	SH3 domain binding	16
	GO:0019901	Protein kinase binding	10
Pathway	hsa05322	Systemic lupus erythematosus	176
	hsa05034	Alcoholism	41
	hsa04110	Cell cycle	12

### 3.4 cis‐regulation of lncRNAs

Through cis‐regulation relationship, 96 DELs were obtained from cis‐regulation analysis, including Pearson P value, Pearson correlation, and cis distance. The Xls format file named “cis of 96 DELs” is uploaded in the Supplementary Materials. In addition, the four most significant cis‐regulation relationships are presented in Figure S1A–D.

### 3.5 lncRNA trans advanced analysis

**FIGURE 5:**
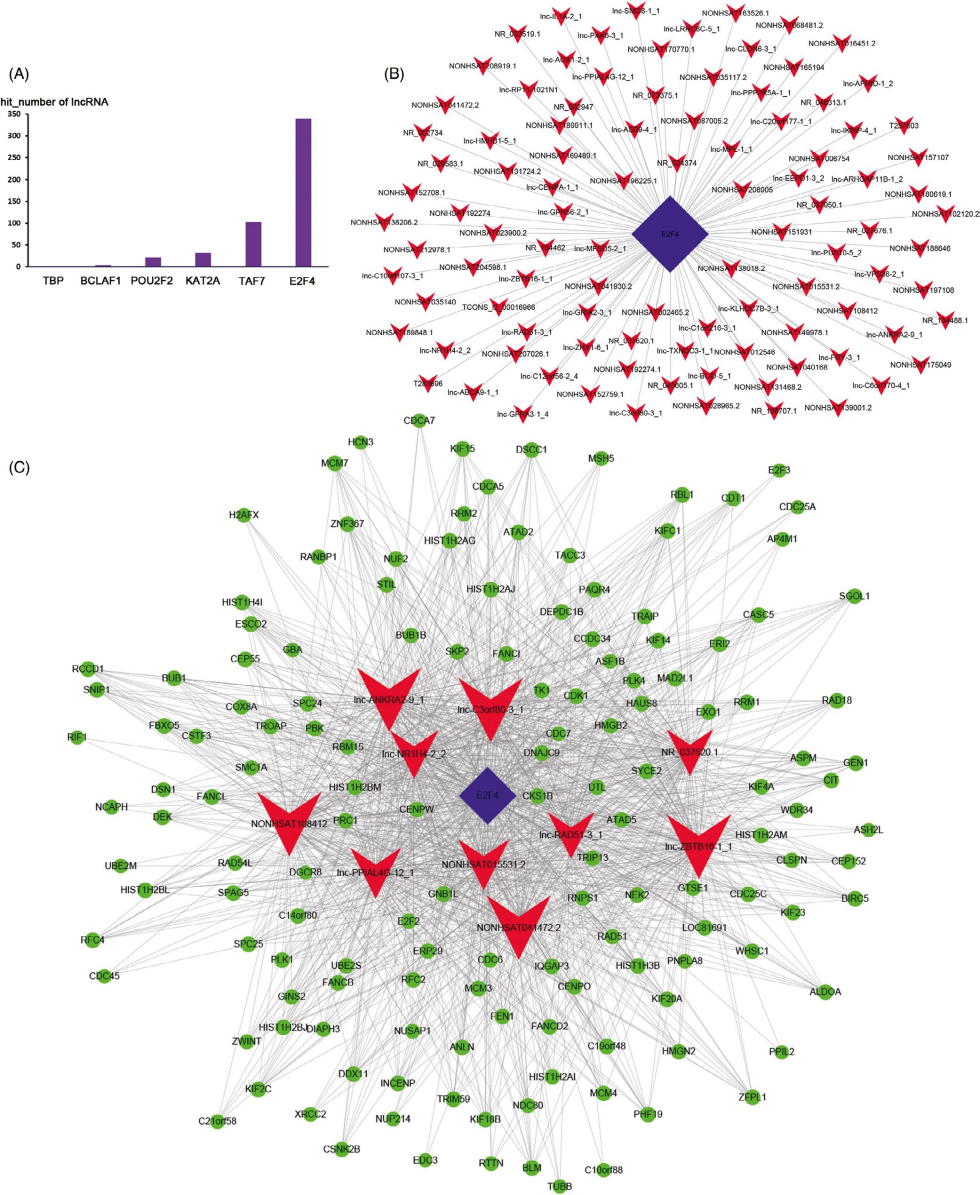
lncRNA trans advanced analysis. (A) The distribution of the top 500 lnc‐trans predictions. (B) The top 100 lncRNA‐TF regulatory network. (C) The top 10 lncRNA‐TF‐mRNA regulatory network. (Blue represents transcript factor E2F4; Red represents lncRNAs; Green represents genes in B and C)

The top 500 lnc‐trans prediction relationships with the highest prediction reliability (sorted by p‐values) were selected, and the frequency of each TF was counted (Figure 5A). The top 100 co‐expression pairs of lncRNA‐TF and top 10 lncRNA‐TF‐mRNA prediction relationships were visualized using Cytoscape to construct the regulatory network (Figure 5B,C). It can be seen that the counts of interactions with E2F4 is the largest, suggesting that E2F4 may be the core transcription factor in the lncRNA‐TF regulatory network in cervical cancer.

## 4 DISCUSSION

An increasing number of lncRNAs have been proven to be molecular regulators of cervical cancer and may provide therapeutic targets. EBIC may act as an oncogenic lncRNA through cooperation with EZH2 and may promote the migration of cervical cancer cells.^20^ CCHE1 binds to PCNA mRNA and promotes its expression, thereby increasing cervical cancer cell proliferation.^21^ HOTAIR not only promotes the proliferation and invasion of cervical cancer cells, but is also related to the recurrence of cervical cancer. In addition, MALAT1, PVT1, H19, and XIST can promote cancer cell proliferation, invasion, and migration and play a role in the progression of cervical cancer.

In this study, 1568 lncRNAs and 2347 mRNAs were found to be differentially expressed between cervical cancer and adjacent noncancerous tissues through microarray analysis. The functional prediction of lncRNAs suggests that both upregulated and downregulated lncRNAs play a role in regulating the mitotic cell cycle and DNA replication by binding to proteins or protein kinases. Transcription factors are a group of protein molecules that can be specifically combined with a specific gene sequence to ensure that the target gene is expressed at a specific time and space with a specific strength. LncRNA‐TF interaction is an important molecular mechanism by which lncRNAs regulate target gene expression. HAND2‐AS1 may inhibit the development of cervical cancer by recruiting E2F4 to the C16orf74 promoter region and down‐regulating the expression of C16orf74.^22^ lncRNA‐trans‐advanced analysis suggested that E2F4 may be the core transcription factor in the lncRNA‐TF regulatory network in cervical cancer. Coincidentally, Kori et al. established and analyzed the human transcriptional regulatory interaction network and identified three TFs that control the transcriptional expression of core cervical cancer genes, namely E2F4, TS1, and CUTL1, of which E2F4 was the most prominent.^23^ Therefore, E2F4 may have potential as a biomarker candidate and therapeutic target.

Owing to the small sample size and lack of in‐depth experiments, these results are relatively superficial. It is necessary to conduct more in‐depth experiments with a larger sample size to clarify the relationship between lncRNAs and the transcription factor E2F4. At the same time, the data from this study will provide some hints to other researchers for further research.

## AUTHOR CONTRIBUTIONS

*Performed the comparative analysis using bioinformatics tools*: Haiyan Yang, Ziyu Fang, and Xiaoli Qu. *Participated in the data analysis and discussion*: Xiaoli Qu and Jiangang Huang. *Analyzed, interpreted the results, and wrote the manuscript*: Haiyan Yang. *Participate and guide data analysis*: Fan Zhang. *Conducts revision, language, and grammar correction*: Bin Zhao. *Organized and supervised the project*: Yifeng Wang and Bin Zhao. Adll authors read and approved the final manuscript.

## Supporting information

Fig S1

Supplementary Material

Supplementary Material

Supplementary Material

## Data Availability

The data used to support the findings of this study are included in the article. All detailed data can be obtained from the author.
