# Supplementary material for: E2F4 may be a core transcription factor in the lncRNA‐TF regulatory network in cervical cancer
Source: J Clin Lab Anal. 2022 Mar 9;36(4):e24322. doi: 10.1002/jcla.24322 (PMC8993607; doi:10.1002/jcla.24322)
Supplement: Supplementary file 1 — Fig S1 [file JCLA-36-e24322-s004.docx]

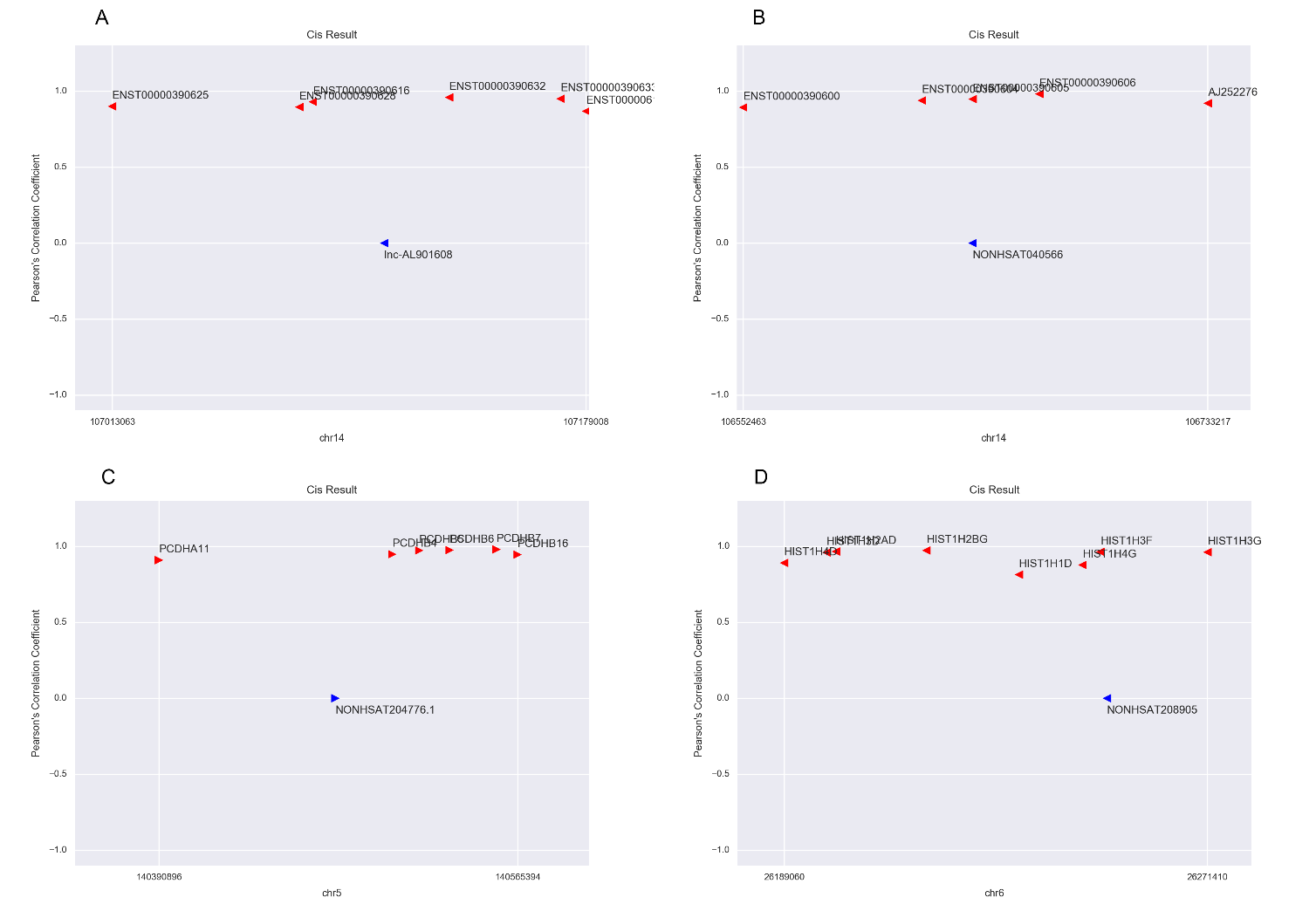


Figure S1:

A: the cis relationship between lnc-AL901608 and ENST00000390633, ENST00000390616, ENST00000390632, ENST00000390628, ENST00000390625, ENST00000617374

B: the cis relationship between NONHSAT040566 and ENST00000390604, ENST00000390606, ENST00000390605, ENST00000390600, AJ252276

C: the cis relationship between NONHSAT204776.1 and PCDHB16, PCDHB5, PCDHB6, PCDHB7, PCDHB4, PCDHA11

D: the cis relationship between NONHSAT208905 HIST1H3F, HIST1H3D,HIST1H1D, HIST1H4G, HIST1H2BG, HIST1H2AD, HIST1H3G, HIST1H4D
